# Extreme climate projections under representative concentration pathways in the Lower Songkhram River Basin, Thailand

**DOI:** 10.1016/j.heliyon.2021.e06146

**Published:** 2021-02-16

**Authors:** Sumana Shrestha, Raywadee Roachanakanan

**Affiliations:** Faculty of Environment and Resource Studies, Mahidol University, Salaya Campus, Nakhon Pathom, 73170, Thailand

**Keywords:** Extreme events, Rainfall, Temperature, Climate change, Thailand

## Abstract

This paper aims to assess changes in the extreme climate indices of the Lower Songkhram River Basin of Thailand under Representative Concentration Pathways (RCPs) scenarios. A linear scaling method was used to correct climate data bias in three Regional Climate Models (RCMs) under RCP 4.5 and RCP 8.5 scenarios. Thereafter, extreme climate indices related to temperature and rainfall were analysed for the wet and dry seasons in upstream and downstream areas of the basin. A total of 14 climate indices were analysed for three time periods: the 2030s (2020–2044), 2055s (2045–2069), and 2080s (2070–2094) and compared with the baseline climate from 1980‒2004. The results show that considerable variability is expected in the extreme climate of the basin in future. The average annual and monthly maximum and minimum temperature is projected to increase, with a lesser increase in the near future and higher in the far future. Heat events (TXx, TXn) are projected to increase while the cold events (TNx, TNn) are projected to decrease in both dry and wet seasons upstream and downstream of the basin. The future average annual rainfall in the basin is projected to decrease under RCP 4.5 and RCP 8.5 scenarios for all three periods. However, the variability in average monthly rainfall is expected to increase in the dry season (Jan–May) and decrease in the wet (Aug–Dec). The most intense rainfall in one day (RX1Day) and five consecutive days (RX5Day) in the wet season is observed to increase in future, with a higher increase in the near future and a lower increase in the far future. The very heavy rainfall days (R20) (the number of days receiving more than 20 mm/day in the basin) are observed to decrease in both wet and dry seasons under RCP 4.5 and RCP 8.5 scenarios in both locations. The results of this study will be helpful for the planning and management of natural resources as well as disaster risk reduction in the Lower Songkhram River Basin.

## 1 Introduction

The Lower Songkhram River Basin (LSRB) in Northeast Thailand has a rich, diverse, and dynamic ecosystem that supports a range of economic activities. The Songkhram River in its lower reaches meanders over a broad floodplain, containing the largest remaining area of seasonally inundated freshwater swamp forest in Thailand, interspersed with converted agricultural land and an array of ponds, reservoirs, channels, swamps and oxbow lakes. These wetlands are not only important sites in their own right for aquatic biodiversity, especially fish species, but also vital for the livelihoods of local people who utilise them and harvest the abundant wetland products found across this region (Kunarat, 2001). The pattern of seasonal flooding and recession, range of natural habitats inundated across the Songkhram floodplain, and the connections between the flow of the Songkhram and Mekong Rivers are fundamental to the productivity of rich natural resources. Any changes to the flooding patterns within the Songkhram River alter habitats and floodplain connectivity, thereby increasing the vulnerability of the ecosystem and local economies (Friend et al., 2006). The Lower Songkhram River Basin also supports rice production for subsistence agriculture and the economy. The majority of farmers grow rainfed rice (~50% of the area) for their livelihoods during the rainy season (May–October).

Although the Lower Songkhram River Basin supports ecosystem services such as wetlands, fisheries, forests, and crop production, it has been under the increasing threat of environmental and climate change over the past decades. Water resources availability, fisheries, and crop production in the basin are very sensitive to changes in climate-related extremes such as maximum and minimum temperature, consecutive dry and wet days, and extreme rainfall. Understanding the potential trends and variability of such future climate extremes is of great importance for the effective planning and management of natural resources as well as disaster risk reduction (Rajbhandaria et al., 2017). Climate extremes are increasingly attracting attention because of their large societal impact on multiple sectors, such as agriculture, economies, and human health (Easterling et al., 2000; Meehl and Tebaldi 2004; Negri et al., 2005; Pereira et al., 2017, 2019). Significant increasing trends in numerous climate extreme indicators have been reported over many regions using a variety of datasets and methods (Frich et al., 2002; Alexander et al., 2006; Ning and Qian, 2009). A study by Chinvanno (2009) reported that the average maximum and minimum temperature in Thailand and the Southeast Asia region will increase in future. The study projected the range of temperature increase in the future to be approximately 2–3 °C during the middle of the century, with increasing trend continuing until the end of the twenty-first century when most parts of the region will be warmer.

Reducing the negative impacts of climate change in water, agriculture, and fisheries sectors in the Lower Songkhram River Basin requires a thorough assessment of the climate change impact and implementation of adaptation strategies. Moreover, as the first step, these activities require collection of future climate data for the basin. General Circulation Models (GCMs) and Regional Climate Models (RCMs) provide future climate projection scenarios and aspects of climate variability and extremes which can be instrumental for impact studies and adaptation planning. However, the climate change scenarios provided by the GCMs and RCMs have a coarse resolution which is insufficient for use by policymakers and planners, and need to be downscaled to derive a finer resolution at local scale by applying the appropriate downscaling techniques. A good understanding of the spatial and temporal distribution of possible changes in extreme events at the local scale is important for sustainable water resources and natural resources planning and management.

This research uses Representative Concentration Pathways (RCPs); new scenarios recommended by the Intergovernmental Panel on Climate Change (IPCC) for climate change impact and adaptation studies (IPCC, 2013). The RCP 4.5 is an intermediate stabilisation scenario which can only be achieved if proper adaptation strategies are implemented. The RCP 8.5 pathway arises when little or no effort is made to reduce greenhouse gas emissions (van Vuuren et al., 2011).

The present study aims to project the changes in extreme temperature and rainfall in the Lower Songkhram River Basin using three selected RCMs from the Coupled Model Intercomparison Project Phase 5 (CMIP5) under two Representative Concentration Pathway (RCP) scenarios, namely RCP 4.5 and RCP 8.5. The temporal and spatial variation of future extreme temperature and rainfall in different areas of the basin are based on the projected annual and seasonal values and the future patterns analysed.

## 2 Data and methods

### 2.1 Study area

**Figure 1:**
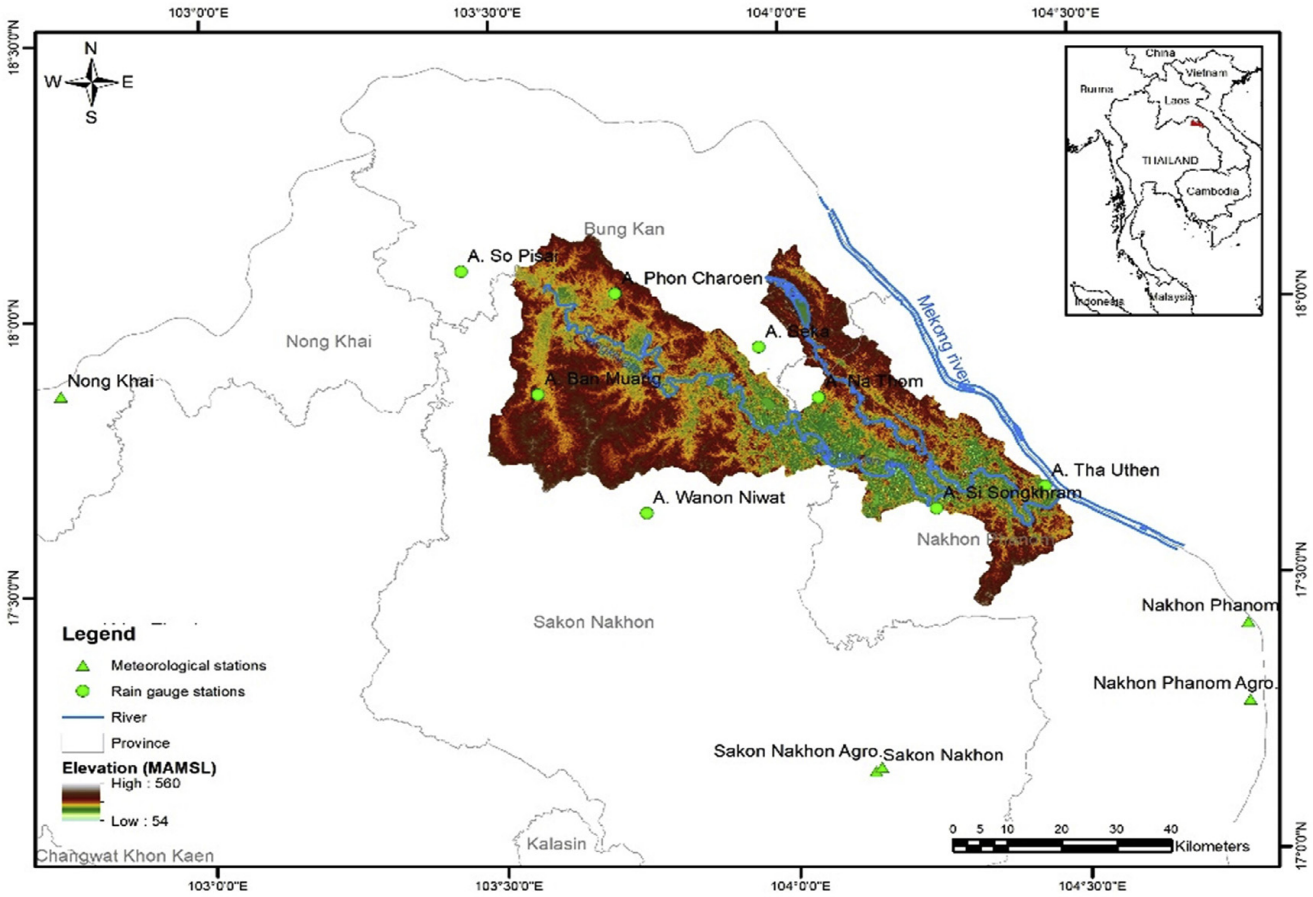
Location map of the Lower Songkhram River Basin in Thailand, together with rainfall and meteorological stations.

The Lower Songkhram River Basin is located in Northeast Thailand, between 17º20’ to 18º10’ N and 103º30’ to 104º30’ E, draining water from 3,049 km^2^ of the area (Figure 1). The basin includes 12 districts in three provinces: Nakhon Phanom, Sakhon Nakhon, and Nong Khai Province. The majority of the basin area consists of flat wetland where the elevation ranges from 145–160 m above mean sea level (masl) with a mild gradient of 1:30 000. These tributaries together with the Lower Songkhram River create the lowland floodplain (Blake, 2012).

The basin has a tropical semi-humid climate with three seasons (rainy, winter, and summer). The basin experiences greater variability during rainfall, with more than 90% of the total annual rainfall occurring from May–October. For example, the southwest part of the study area receives less than 1450 mm/year of rainfall, while in the northeast region of the basin this increases to more than 2450 mm/year. Monthly relative humidity ranges from 65–78%, depending on the season and time of the day. For the period from 1995–2009, the minimum and maximum air temperatures in the basin are observed as 19 °C and 34 °C, respectively with a mean annual air temperature of around 27 °C and an average annual evaporation rate of between 1200–1900 mm/year (Muangthong et al., 2012).

The major land use in the basin consists of paddy field followed by forest. The majority of farmers grow rainfed rice during May–October, while a small percentage grow irrigated rice (December–April). The local economy is heavily dependent on products originating from seasonal forest floods, including fresh and fermented fish, wild plants, cultivated vegetables, and large livestock. The number of families owning cattle or buffalo has steadily increased, while the number of animals per household has decreased due to communal grazing plains often being occupied by agribusiness ventures.

### 2.2 Observed climate data

Historical data from eight rainfall and six meteorological stations inside the basin and nearby was collected from the Thai Meteorological Department (TMD) (Figure 1). The location of the stations, elevation data, and average annual rainfall for each station are presented in Table 1. Observed data from 1980–2004 was also considered as the baseline period for climate change analysis in this study.

**Table 1:** List of rainfall and meteorological stations in the Lower Songkhram River Basin.

SN	Station Name	Province	Station Type	Latitude	Longitude
1	A. Phon Charoen	Bung Kan	Rain gauge	18° 02′	103° 43′
2	A. Seka	Bung Kan	Rain gauge	17° 53′	103° 58′
3	A. So Pisai	Bung Kan	Rain gauge	18° 06′	103° 23′
4	A. Ban Muang	Sakon Nakhon	Rain gauge	17° 48′	103° 35′
5	A. Wanon Niwat	Sakon Nakhon	Rain gauge	17° 39′	103° 46′
6	A. Na Thom	Nakhon Phanom	Rain gauge	17° 47′	104° 06′
7	A. Si Songkhram	Nakhon Phanom	Rain gauge	17° 37′	104° 18′
8	A. Tha Uthen	Nakhon Phanom	Rain gauge	17° 31′	104° 36′
9	Nakhon Phanom	Nakhon Phanom	Meteorology	17° 25′	104° 47′
10	Nakhon Phanom Agromet	Nakhon Phanom	Meteorology	17° 26′	104° 47′
11	Nong Khai	Nong Khai	Meteorology	17° 52′	102° 44′
12	Sakon Nakhon	Sakon Nakhon	Meteorology	17° 09′	104° 08′
13	Sakon Nakhon Agromet	Sakon Nakhon	Meteorology	17° 07′	104° 03′
14	Udon Thani	Udon Thani	Meteorology	17° 23′	102° 48′

### 2.3 Future climate data

Future climate data for three RCMs: ACCESS1-CSIRO-CCAM, CNRM-CM5-CSIRO-CCAM, and MPI-ESM-LR-CSIRO-CCAM was used to project future climate extremes. This data was downloaded from the Coordinated Regional Climate Downscaling Experiment in East Asia (CORDEX EAST ASIA) website (retrieved from https://cordex-ea.climate.go.kr on 10 April 2017) under RCP 4.5 and RCP 8.5 climate scenarios. Details of the three RCMs are presented in Table 2. Several climate change studies in Thailand have evaluated the above three RCMs and found them suitable for use in impact assessment studies (Boonwichai et al., 2018; 2019; Shrestha et al., 2017a, b). Therefore, in this study, the same RCMs are also selected for the projection of future climate in the Songkhram River Basin.

**Table 2:** Selected Regional Circulation Models (RCMs) used in this study.

SN	RCMs	Institute	Resolution (latitude x longitude)	Driving GCM
1	ACCESS1-CSIRO-CCAM	Commonwealth Scientific and Industrial Research Organisation (CSIRO)	0.5° x 0.5°	ACCESS1.0 The Australian Community Climate and Earth System Simulator Coupled Model
2	CNRM-CM5-CSIRO-CCAM	Commonwealth Scientific and Industrial Research Organisation (CSIRO)	0.5° x 0.5°	CNRM-CM5 Le Centre National de Recherches Météorologiques
3	MPI-ESM-LR –CCAM	Commonwealth Scientific and Industrial Research Organisation (CSIRO)	0.5° x 0.5°	MPI-ESM-LR The Max-Planck-Institut für Meteorologie - Earth System Model running on low resolution grid

### 2.4 Development of extreme indices

The joint CCl/CLIVAR/JCOMM Expert Team (ET) on Climate Change Detection and Indices (ETCCDI) has identified 27 core indices (11 for precipitation and 16 for temperature) for the study of extreme climate events (Alexander et al., 2006; http://etccdi.pacificclimate.org/list_27_indices. shtml). These indices have been widely used in the detection, attribution, and projection of changes in extreme climate (Alexander et al., 2006; Min et al., 2011; Orlowsky and Seneviratne, 2012; Donat et al., 2013; Wen et al., 2013; Xu et al., 2013; Bartolomeu et al., 2016; Fonseca et al., 2016; Viceto et al., 2019). Sillmann et al. (2011) compared the ETCCDI computed from observations and model simulations with the Coupled Model Intercomparison Project Phase 5 (CMIP5; Taylor et al., 2012) and found that CMIP5 models were generally able to reproduce the historical trend patterns of these climate extreme indices. Seven indices relating to temperature and another seven to rainfall were selected for this study (Table 3).

**Table 3:** Selected indices relating to temperature and rainfall in the LSRB (historical: 1980–2004 and future: 2011–2100).

Indices	Notation	Unit	Definition
**Temperature**			
Maximum daily maximum temperature	TXx	°C	Monthly maximum value of daily maximum temperature
Maximum daily minimum temperature	TNx	°C	Monthly maximum value of daily minimum temperature
Minimum daily maximum temperature	TXn	°C	Monthly minimum value of daily maximum temperature
Minimum daily minimum temperature	TNn	°C	Monthly minimum value of daily minimum temperature
Warm nights	TN90p	%	Percentage of days when TN > 90th percentile
Warm days	TX90p	%	Percentage of days when TX > 90th percentile
Warm spell duration index	WSDI	days	Annual count of days with at least 6 consecutive days when TX > 90th percentile
**Rainfall**			
Consecutive dry days	CDD	days	Maximum number of consecutive days with daily precipitation <1 mm
Consecutive wet days	CWD	days	Maximum number of consecutive days with daily precipitation ≥1 mm
Annual total wet-day precipitation	PRCPTOT	mm	Annual total precipitation in wet days (daily precipitation ≥1 mm)
Very wet days	R95p	mm	Annual total PRCP when RR > 95p
Monthly maximum one-day precipitation	RX1day	mm	Most intense rainfall event in one day for a given month
Monthly maximum consecutive five-day precipitation	RX5day	mm	Most intense rainfall event in five consecutive days for a given month
Heavy rainfall days	R20	days	Annual count of days when precipitation >20 mm

### 2.5 Bias correction

**Figure 2:**
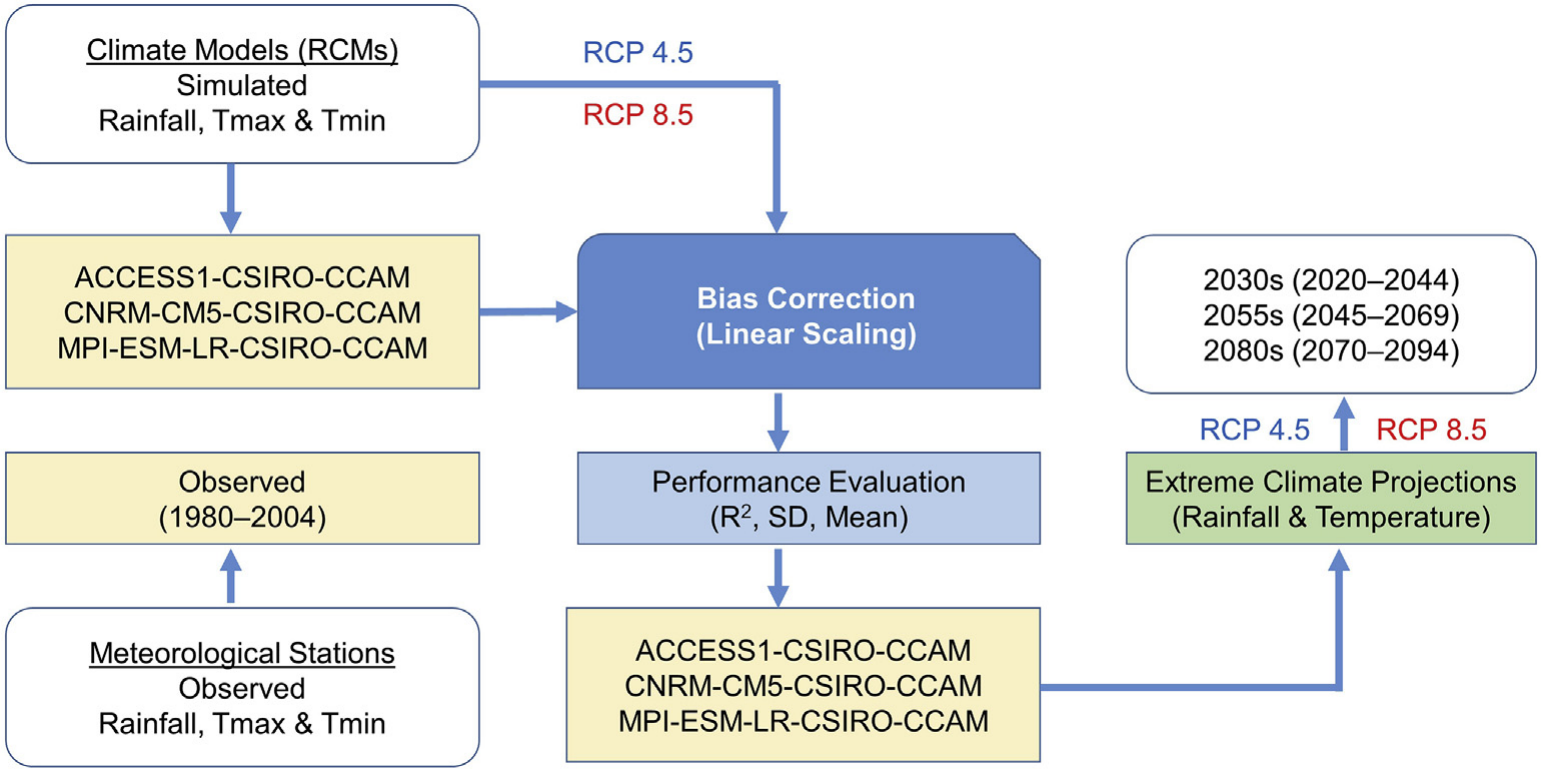
Overall methodology adopted to project extreme climate in the study area.

Due to RCM bias, Teutschbein and Seibert (2010) recommended employing bias correction techniques, even though they can add significantly to uncertainties in impact studies on climate change. In the related literature, several studies have compared the different approaches used to minimise biases. According to Shrestha et al. (2017a, b), the linear downscaling technique (Lenderink et al., 2007), which runs with monthly correction values in accordance with the differences between simulated and present-day measured values, is sufficiently capable of correcting bias from RCM outputs in comparison to the quantile mapping technique. Therefore, the linear downscaling technique was chosen for this study since it is the simplest and has been utilised in several previous studies (Ines and Hansen, 2006; Shrestha et al., 2017a, b; Teutschbein and Seibert, 2012). The overall methodology adopted in this study is illustrated in Figure 2. In the linear scaling method, the difference between monthly observed and monthly simulated values is used to correct the biases. The difference is then applied to the simulated climate data to obtain bias corrected climate data for the basin. The following equations are used in the linear scaling bias correction method:

(1) $$P_{his}{\left(d\right)}^{*}=P_{his}\left(d\right).\left[\mu_{m}\left\{P_{obs}\left(d\right)\right\}/\mu_{m}\left\{P_{his}\left(d\right)\right\}\right]$$

(2) $$P_{sim}{\left(d\right)}^{*}=P_{sim}\left(d\right).\left[\mu_{m}\left\{P_{obs}\left(d\right)\right\}/\mu_{m}\left\{P_{his}\left(d\right)\right\}\right]$$

(3) $$T_{his}{\left(d\right)}^{*}=T_{his}\left(d\right)+\left[\mu_{m}\left\{T_{obs}\left(d\right)\right\}-\mu_{m}\left\{T_{his}\left(d\right)\right\}\right]$$

(4) $$T_{sim}{\left(d\right)}^{*}=T_{sim}\left(d\right)+\left[\mu_{m}\left\{T_{obs}\left(d\right)\right\}-\mu_{m}\left\{T_{his}\left(d\right)\right\}\right]$$

where *P* is the precipitation; *T* is the temperature; *d* is the daily time-series; *μ*_*m*_ is the long-term monthly mean; an asterisk (∗) is bias corrected; *his* is the historical raw RCM data; *obs* is the observed data; and *sim* is the raw RCM future data.

Performance of the bias correction method was evaluated using four statistical indicators: standard deviation (SD) and mean values of temperature and rainfall. The performance was evaluated using daily data from 1980–2004 for six temperature stations and eight rain gauge stations in three RCMs. Future climate extremes were projected for three future periods: the 2030s (2020–2044), 2055s (2045–2069), and 2080s (2070–2094) and compared with the baseline climate (1980–2004. Projected changes in the annual and seasonal indices are summarised using box-and-whisker plots. These plots consist of the multimodel median, interquartile model spread (the range between the 25th and 75th quantiles box), and the full intermodel range (whiskers). The spatial pattern of change was examined by dividing the basin into upstream and downstream confluence points of the Lower Songkhram River.

## 3 Results and discussion

### 3.1 Performance evaluation of bias correction

The linear scaling method shows a satisfactory performance in correcting the bias in RCM data. The performance was evaluated using daily data from 1980–2004 for six temperature stations and eight rain gauge stations in three RCMs. The results indicate that the standard deviation and average maximum and minimum temperatures of the corrected RCMs are similar to the observed data for all meteorological stations (Tables 4 and 5). Similarly, the average rainfall and standard deviation of corrected RCMs are closer to the observed rainfall data for all rain gauge stations (Table 6). For example, the mean and SD observed temperatures are 31.7 and 3.1 °C, respectively at Nakhon Phanom station. However, the mean was lower and the SD higher in temperature when simulated by all three RCMs. After bias correction, the mean value was equal to the mean of observed temperature and the SD reduced. This shows a better bias correction performance in the corresponding stations. Similar results were also obtained for all the other stations.

**Table 4:** Comparison of the mean and SD in maximum temperature between RCM and observed data.

Station		ACCESS1-CSIRO-CCAM		CNRM-CM5-CSIRO-CCAM		MPI-ESM-LR-CSIRO-CCAM	
		Mean	SD (°C)	Mean	SD (°C)	Mean	SD (°C)
Nakhon Phanom	Obs	31.7	3.1	31.7	3.1	31.7	3.1
	RCM His	29.1	5.5	29.0	5.6	29.4	5.4
	RCM Corr His	31.7	4.6	31.7	4.5	31.7	4.6
Nakhon Phanom Agromet	Obs	30.8	3.2	30.8	3.2	30.8	3.2
	RCM His	29.1	5.5	29.0	5.6	29.4	5.4
	RCM Corr His	30.8	4.6	30.8	4.5	30.8	4.6
Nong Khai	Obs	32.2	3.3	32.2	3.3	32.2	3.3
	RCM His	31.3	4.0	31.1	4.1	31.3	4.0
	RCM Corr His	32.2	3.8	32.2	3.8	32.2	3.8
Sakon Nakhon	Obs	31.6	3.2	31.6	3.2	31.6	3.2
	RCM His	31.0	4.3	30.8	4.4	31.1	4.3
	RCM Corr His	31.6	4.0	31.6	3.9	31.6	4.0
Sakon Nakhon Agromet	Obs	31.7	3.1	31.7	3.1	31.7	3.1
	RCM His	31.0	4.3	30.8	4.3	31.1	4.2
	RCM Corr His	31.7	3.9	31.7	3.8	31.7	3.8
Udon Thani	Obs	32.4	3.2	32.4	3.2	32.4	3.2
	RCM His	31.3	4.0	31.1	4.1	31.3	4.0
	RCM Corr His	32.4	3.8	32.4	3.9	32.4	3.8

**Table 5:** Comparison of the mean and SD in minimum temperature between RCM and observed data.

Station		ACCESS1-CSIRO-CCAM		CNRM-CM5-CSIRO-CCAM		MPI-ESM-LR-CSIRO-CCAM	
		Mean	SD (°C)	Mean	SD (°C)	Mean	SD (°C)
Nakhon Phanom	Obs	21.6	3.8	21.6	3.8	21.6	3.8
	RCM His	20.4	4.1	20.3	4.3	20.4	4.1
	RCM Corr His	21.6	4.0	21.6	4.0	21.6	4.1
Nakhon Phanom Agromet	Obs	20.6	4.2	20.6	4.2	20.6	4.2
	RCM His	20.4	4.1	20.3	4.3	20.4	4.1
	RCM Corr His	20.6	4.4	20.6	4.4	20.6	4.4
Nong Khai	Obs	21.9	3.7	21.9	3.7	21.9	3.7
	RCM His	21.9	4.4	21.7	4.6	21.9	4.5
	RCM Corr His	21.9	4.1	21.9	4.1	21.9	4.1
Sakon Nakhon	Obs	21.9	4.0	21.9	4.0	21.9	4.0
	RCM His	21.7	4.5	21.5	4.7	21.7	4.5
	RCM Corr His	21.9	4.3	21.9	4.3	21.9	4.3
Sakon Nakhon Agromet	Obs	20.9	4.3	20.9	4.3	20.9	4.3
	RCM His	21.9	4.2	21.7	4.4	21.8	4.2
	RCM Corr His	20.9	4.5	20.9	4.5	20.9	4.5
Udon Thani	Obs	22.0	3.8	22.0	3.8	22.0	3.8
	RCM His	21.9	4.4	21.7	4.6	21.9	4.5
	RCM Corr His	22.0	4.1	22.0	4.2	22.0	4.2

**Table 6:** Comparison of the mean and SD in rainfall between RCM and observed data.

Station		ACCESS1-CSIRO-CCAM		CNRM-CM5-CSIRO-CCAM		MPI-ESM-LR-CSIRO-CCAM	
		Mean	SD (mm)	Mean	SD (mm)	Mean	SD (mm)
A. Phon Charoen	Obs	1810	171	1810	171	1810	171
	RCM His	620	51	656	65	639	56
	RCM Corr His	1810	185	1810	196	1810	185
A. Seka	Obs	1901	159	1901	159	1901	159
	RCM His	620	51	656	65	639	56
	RCM Corr His	1901	187	1901	199	1901	189
A. So Pisai	Obs	1665	137	1665	137	1665	137
	RCM His	706	63	761	77	724	67
	RCM Corr His	1665	165	1665	184	1665	168
A. Ban Muang	Obs	1851	161	1851	161	1851	161
	RCM His	615	46	650	60	621	47
	RCM Corr His	1851	171	1851	184	1851	170
A. Wanon Niwat	Obs	1450	119	1450	119	1450	119
	RCM His	620	51	656	65	639	56
	RCM Corr His	1450	140	1450	148	1450	140
A. Na Thom	Obs	1452	116	1452	116	1452	116
	RCM His	620	51	656	65	639	56
	RCM Corr His	1452	138	1452	144	1452	137
A. Si Songkhram	Obs	1578	131	1578	131	1578	131
	RCM His	604	48	639	60	610	54
	RCM Corr His	1578	148	1578	162	1578	161
A. Tha Uthen	Obs	2295	203	2295	203	2295	203
	RCM His	585	56	600	61	551	49
	RCM Corr His	2295	244	2295	249	2295	238

### 3.2 Future projections

The future projections of extreme temperature and rainfall in the dry and wet seasons were analysed for three periods: the 2030s (2020–2044), 2055s (2045–2069), and 2080s (2070–2094) and compared with the baseline climate (1980–2004).

#### 3.2.1 Maximum and minimum temperature projection

**Figure 3:**
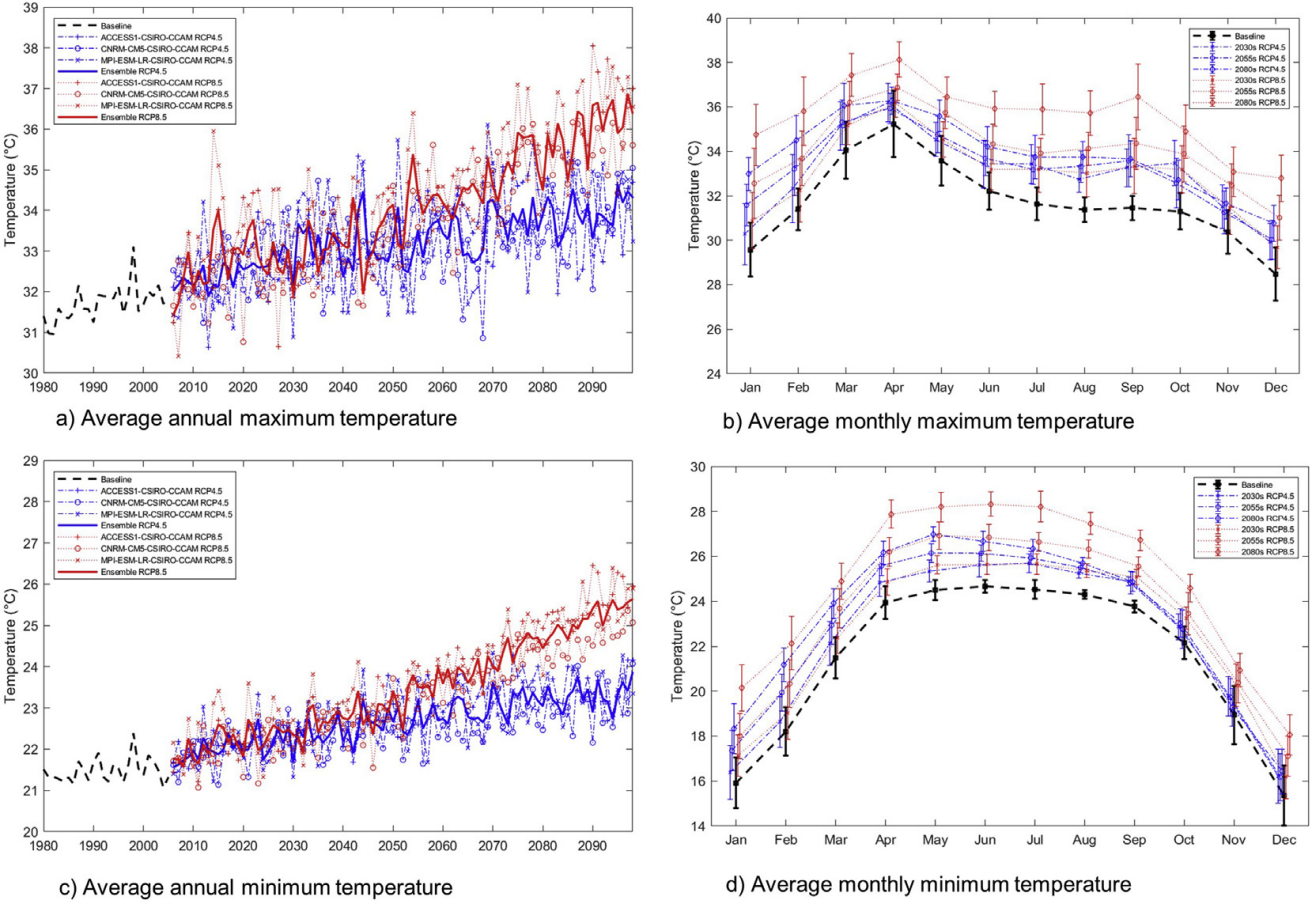
(a) projected future average annual maximum temperature, (b) average monthly maximum temperature, (c) average annual minimum temperature, and (d) average monthly minimum temperature under RCP 4.5 and RCP 8.5 scenarios in the Lower Songkhram River Basin.

The average annual and monthly maximum and minimum temperatures in the basin are projected to increase in future, with a lesser increase in the near future and a higher increase in the far future under both RCP 4.5 and RCP 8.5 scenarios (Figure 3). Table 7 shows the average values and corresponding change in temperature compared to the baseline period. The baseline (1980–2004) average annual maximum and minimum temperature are 31.7 and 21.5 °C, respectively. The average annual maximum temperature is projected to increase by 1.3, 1.5, and 2.1 °C under RCP 4.5 scenario, while the average annual minimum temperature is projected to increase by 1.3, 2.4, and 3.9 °C under RCP 8.5 scenario for the 2030s, 2055s, and 2080s, respectively. Similarly, the future average annual and monthly minimum temperatures are also projected to increase. The average annual minimum temperature is projected to increase by 0.8, 1.3, and 1.7 °C under RCP 4.5 scenario and by 1.0, 2.0, and 3.3 °C under RCP 8.5 for the 2030s, 2055s, and 2080s, respectively. The average monthly maximum and minimum temperatures are also projected to increase in all months compared to the baseline period (Figure 3). The magnitude of increment under the RCP 8.5 scenario is higher than for RCP 4.5. Previous studies by Boonwichai et al. (2018; 2019), Shrestha et al. (2017a, b), Babel et al. (2011), and Chinvanno and the Southeast Asia START Regional Center (2009) also reported similar results, indicating that temperatures in Thailand could increase by up to 2–3 °C by 2100.

**Table 7:** Projected average annual maximum and minimum temperatures and their corresponding changes under RCP 4.5 and RCP 8.5 scenarios for three future periods in the LSRB.

Temperature	Period	RCP 4.5	Change	RCP 8.5	Change
Maximum Temperature (°C)	Baseline = 31.7				
	2030s	33.0	+1.3	33.0	+1.3
	2055s	33.2	+1.5	34.1	+2.4
	2080s	33.8	+2.1	35.6	+3.9
Minimum Temperature (°C)	Baseline = 21.5				
	2030s	22.3	+0.8	22.5	+1.0
	2055s	22.8	+1.3	23.5	+2.0
	2080s	23.2	+1.7	24.8	+3.3

#### 3.2.2 Rainfall projection

**Figure 4:**
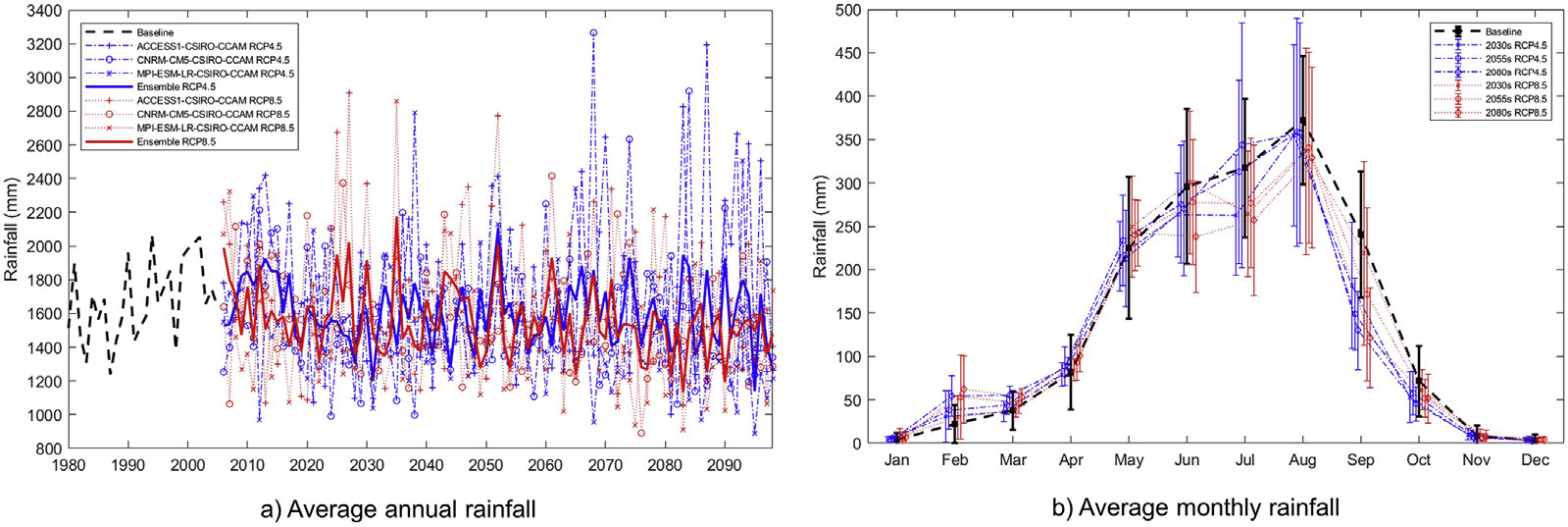
(a) projected future average annual rainfall and (b) average monthly rainfall under RCP 4.5 and RCP 8.5 scenarios for the Lower Songkhram River Basin.

The average annual rainfall in the basin for the baseline period (1980–2004) is 1680 mm. However, the average annual rainfall in the basin is projected to decrease in future under RCP 4.5 and RCP 8.5 scenarios for all three periods (Figure 4a). The rate of decrease is higher (-195 mm/yr) in the near future and lower (-107 mm/yr) in the far future under the RCP 4.5 scenario in contrast to RCP 8.5 (Table 8). However, the average monthly rainfall is expected to vary in future. The average monthly rainfall is expected to increase in the dry season (Jan–May) and decrease in the wet season (Aug–Dec) (Figure 4b).

**Table 8:** Projected average annual rainfall and corresponding changes under RCP 4.5 and RCP 8.5 scenarios for three future periods in the LSRB.

Period		Average annual rainfall (mm)			
		RCP 4.5	Change	RCP 8.5	Change
Baseline (1980–2004)	1680				
2030s (2020–2044)		1495	-195	1606	-74
2055s (2045–2069)		1575	-115	1568	-112
2080s (2070–2094)		1573	-107	1474	-206

Previous studies also report that future rainfall may increase or decrease in many parts of Thailand (Boonwichai et al., 2018; 2019; Shrestha et al., 2017a, b; Arunrat and Pumijumnong, 2015; Babel et al., 2011; Chinvanno and Center, 2009).

#### 3.2.3 Projection of temperature extremes

**Figure 5:**
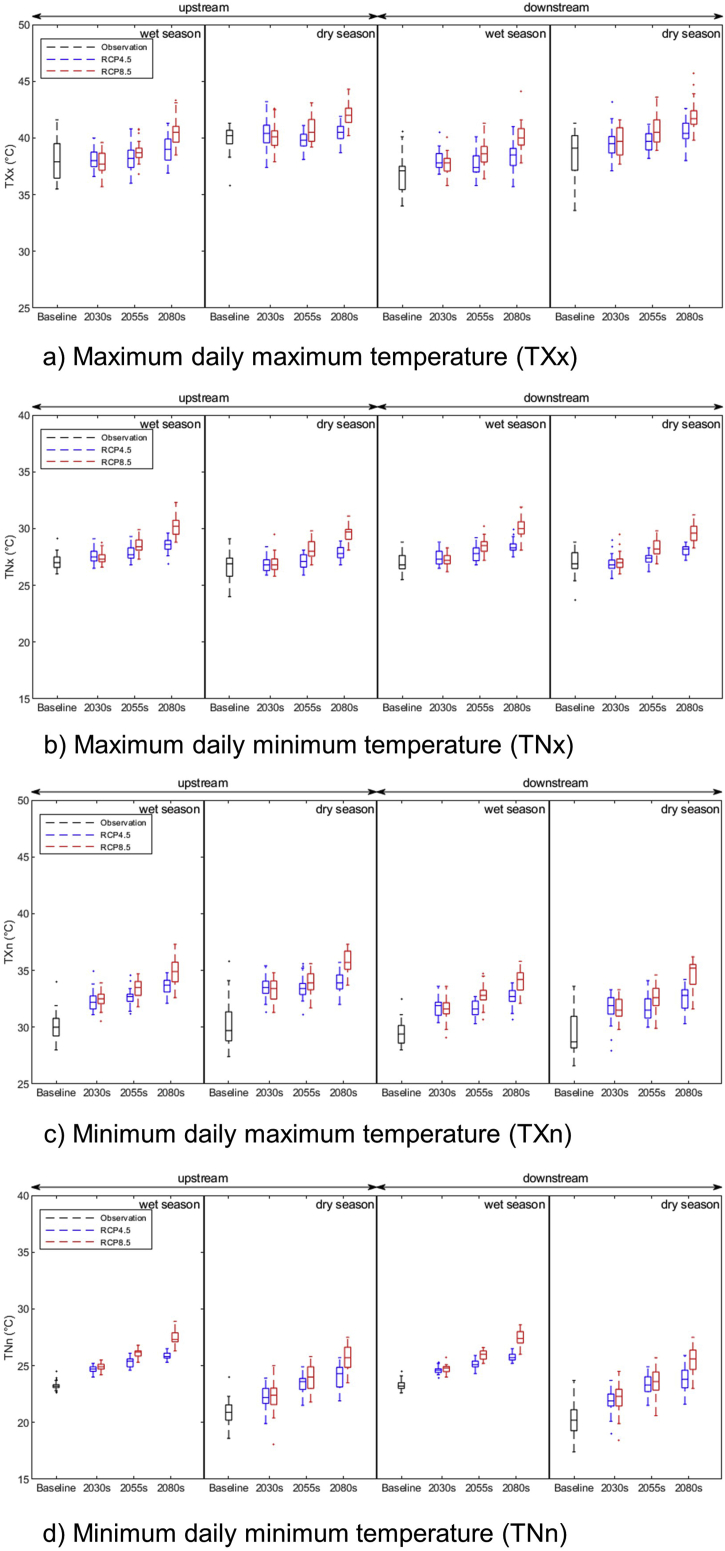
(a) projected maximum daily temperature, (b) maximum daily minimum temperature, (c) minimum daily maximum temperature, and (d) minimum daily minimum temperature upstream and downstream in the LSRB during wet and dry seasons under RCP4.5 and RCP8.5 scenarios. Boxes indicate the interquartile model spread (25th and 75th quantiles) with the horizontal line indicating the ensemble median and the whiskers showing the extreme range of an ensemble of three RCMs.

Extreme indices such as maximum daily maximum temperature (TXx) and maximum daily minimum temperature (TNx) are considered as heat events, while minimum daily maximum temperature (TXn) and minimum daily minimum temperature (TNn) are considered as cold events. These indices were analysed upstream and downstream of the basin for both dry and wet seasons. Heat events are projected to increase while cold events are projected to decrease in both dry and wet seasons upstream and downstream of the basin (Figure 5).

During the wet season, the maximum daily maximum temperature (TXx) is expected to increase by 1.1 and 2.5 °C upstream and by 1.5 and 3.3 °C downstream under RCP 4.5 and RCP 8.5 scenarios, respectively in the 2080s. In the dry season, the maximum daily maximum temperature is expected to increase by 0.5 and 2.1 °C upstream and by 1.8 and 3.3 °C downstream under RCP 4.5 and RCP 8.5 scenarios, respectively in the 2080s (Table 9). Similarly, during the wet season, the minimum daily minimum temperature (TNn) is expected to increase by 2.6 and 4.2 °C upstream and by 2.5 and 4.1 °C downstream under RCP 4.5 and RCP 8.5 scenarios, respectively in the 2080s. In the dry season, the minimum daily minimum temperature is expected to increase by 3.1 and 4.8 °C upstream and by 3.5 and 5.3 °C downstream under RCP 4.5 and RCP 8.5 scenarios, respectively in the 2080s (Supplementary Table). It can be observed that the basin is expected to experience a higher rate of increase in minimum temperature compared to maximum temperature in the future.

**Table 9:** Comparison of future extreme temperature indices with the baseline period during wet and dry seasons upstream and downstream of the LSRB. The value represents an ensemble of three RCMs.

Indices	Period	Upstream				Downstream			
		Wet season		Dry season		Wet season		Dry season	
		RCP4.5	RCP8.5	RCP4.5	RCP8.5	RCP4.5	RCP8.5	RCP4.5	RCP8.5
TXx (°C)	Baseline	38.0		39.9		36.9		38.7	
	2030s	38.2	37.9	40.4	40.1	38.1	37.8	39.6	39.7
	change	0.2	-0.1	0.4	0.1	1.2	0.9	0.9	1.0
	2055s	38.2	38.8	39.8	40.7	37.7	38.7	39.7	40.7
	change	0.3	0.8	-0.1	0.7	0.8	1.9	1.0	2.0
	2080s	39.1	40.5	40.4	42.0	38.4	40.1	40.5	42.0
	change	1.1	2.5	0.5	2.1	1.5	3.3	1.8	3.3
TNx (°C)	Baseline	27.1		26.7		27.0		27.0	
	2030s	27.5	27.4	26.8	27.0	27.5	27.3	27.0	27.1
	change	0.4	0.3	0.2	0.3	0.4	0.2	-0.1	0.1
	2055s	27.8	28.5	27.1	28.2	27.8	28.5	27.3	28.3
	change	0.7	1.4	0.5	1.5	0.8	1.5	0.3	1.3
	2080s	28.5	30.2	27.8	29.5	28.4	30.0	28.1	29.6
	change	1.4	3.1	1.1	2.8	1.4	3.0	1.1	2.6
TXn (°C)	Baseline	30.1		30.4		29.5		29.5	
	2030s	32.4	32.4	33.5	33.3	31.7	31.6	31.7	31.5
	change	2.3	2.3	3.1	2.9	2.2	2.1	2.2	2.0
	2055s	32.6	33.4	33.5	33.9	31.6	32.8	31.7	32.6
	change	2.5	3.3	3.1	3.6	2.1	3.3	2.3	3.1
	2080s	33.6	34.9	34.0	35.7	32.7	34.1	32.5	34.6
	change	3.5	4.8	3.6	5.4	3.2	4.6	3.1	5.1
TNn (°C)	Baseline	23.2		20.9		23.3		20.3	
	2030s	24.7	24.9	22.2	22.3	24.6	24.7	21.9	22.0
	change	1.5	1.7	1.4	1.4	1.3	1.4	1.6	1.7
	2055s	25.3	26.1	23.4	23.9	25.1	25.9	23.3	23.6
	change	2.1	2.8	2.6	3.1	1.8	2.7	3.0	3.3
	2080s	25.8	27.5	24.0	25.7	25.8	27.4	23.8	25.6
	change	2.6	4.2	3.1	4.8	2.5	4.1	3.5	5.3
TN90p (%)	Baseline	8.0		8.4		8.3		8.8	
	2030s	16.0	0.7	18.8	6.8	14.0	1.0	19.4	6.4
	change	8.0	-7.2	10.4	-1.6	5.7	-7.3	10.6	-2.4
	2055s	24.6	6.2	26.9	17.9	24.2	6.7	26.2	19.0
	change	16.6	-1.7	18.5	9.5	15.9	-1.6	17.4	10.2
	2080s	54.2	79.9	51.7	66.6	56.8	73.2	52.7	67.2
	change	46.3	72.0	43.3	58.2	48.5	64.9	44.0	58.4
TX90p (%)	Baseline	8.4		8.7		8.5		8.8	
	2030s	25.8	6.1	24.2	6.8	29.6	7.2	27.0	8.7
	change	17.4	-2.3	15.5	-1.9	21.1	-1.3	18.2	0.0
	2055s	28.1	14.2	26.9	19.9	28.4	15.8	26.5	23.2
	change	19.7	5.8	18.2	11.1	19.9	7.3	17.7	14.4
	2080s	44.9	74.8	46.2	68.3	39.1	73.9	45.1	65.4
	change	36.5	66.4	37.5	59.6	30.5	65.4	36.4	56.6
WSDI (days)	Baseline	0		5		0		6	
	2030s	0	0	4	1	0	0	4	2
	change	0	0	-2	-5	0	0	-2	-4
	2055s	0	0	4	3	0	0	4	3
	change	0	0	-1	-2	0	0	-1	-3
	2080s	0	0	10	28	0	0	7	22
	change	0	0	5	23	0	0	2	17

The maximum daily minimum temperature (TXn) and minimum daily maximum temperature (TNx) are also likely to increase in future during both seasons at the two locations. In the downstream areas, daily minimum temperature is expected to increase at a higher rate than in the upstream location. It may increase by 5.1 °C under the RCP 8.5 scenario in the 2080s. The minimum daily maximum temperature is likely to show a greater increase in the upstream location during the wet season. It may increase by 3.1 under the RCP8.5 scenario in the 2080s (Table 9).

**Figure 6:**
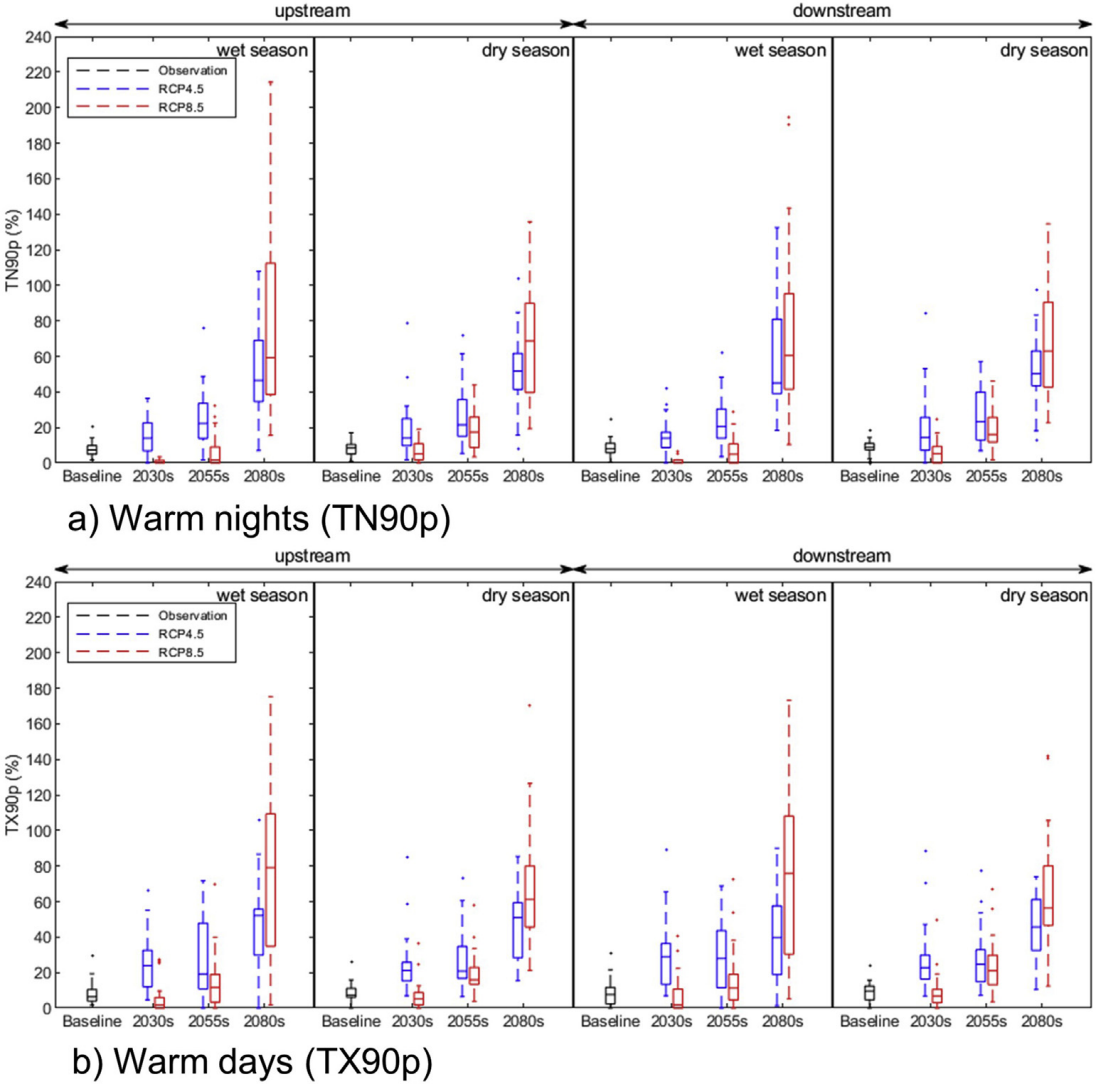
(a) projected warm nights and (b) warm days upstream and downstream of the LSRB during wet and dry season under RCP4.5 and RCP8.5 scenarios.

The warm nights (TN90p) and warm days (TX90p) in both upstream and downstream areas of the basin are projected to increase during both seasons under RCP 4.5 and RCP 8.5 scenarios in future. The rate of increase is greater in the wet season compared to the dry. A lower rate of increase can be observed in the near future and higher in the far future (Figure 6). The increase in heat events and decrease in cold events in the basin might impact several ecosystems services, especially crop production.

#### 3.2.4 Projection of extreme rainfall

**Figure 7:**
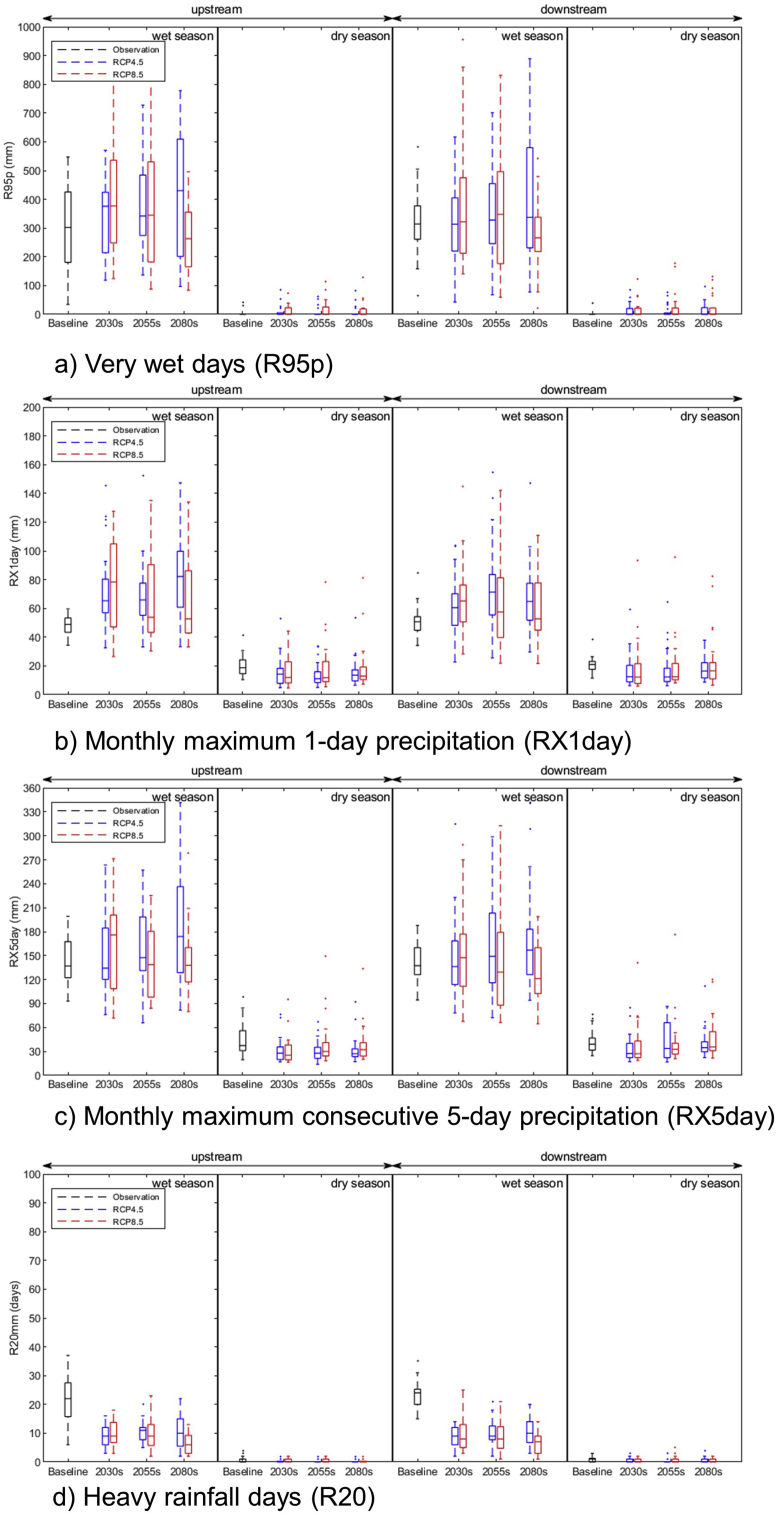
(a) projected very wet days, (b) monthly maximum one-day rainfall, (c) monthly consecutive five-day rainfall, (d) heavy rainfall days upstream and downstream of the LSRB during wet and dry seasons for the 2030s, 2055s, and 2080s under RCP4.5 and RCP8.5 scenarios.

The projected very wet days (R95p), monthly maximum one-day rainfall (RX1day), monthly consecutive five-day rainfall (RX5day), and heavy rainfall days (R20) upstream and downstream of the LSRB during the wet and dry seasons are presented in Figure 7.

The most intense rainfall in one day (RX1Day) in the basin is expected to increase during the wet season and decrease in the dry (Figure 7b). The baseline value of approximately 48–51 mm is expected to rise to 60–83 mm in the wet season and reduce from 20–21 mm to 16–18 mm in the dry season. The value of the most intense rainfall event in five consecutive days (RX5Day) during the wet season is projected to increase in future, with a greater increase in the near future and a lower increase in the far future (Figure 7c). The baseline value of approximately 142 mm is expected to increase to 149–173 mm in the near future. In the dry season, the event is expected to decrease, with a higher rate of decrease in the near future and a lower rate of decrease in the far future (Figure 7c). The baseline value of 42 mm is expected to reduce to 31–38 mm in the near future (Supplementary Table).

Very heavy rainfall days (R20) (the number of days receiving greater than 20 mm/day in the basin) are projected to decrease in both wet and dry seasons under both RCP 4.5 and RCP 8.5 scenarios at the two locations. The baseline value of 22–23 days is expected to reduce to 6–11 days in the far future. In the dry season, no very heavy rainfall days are expected in the future.

**Figure 8:**
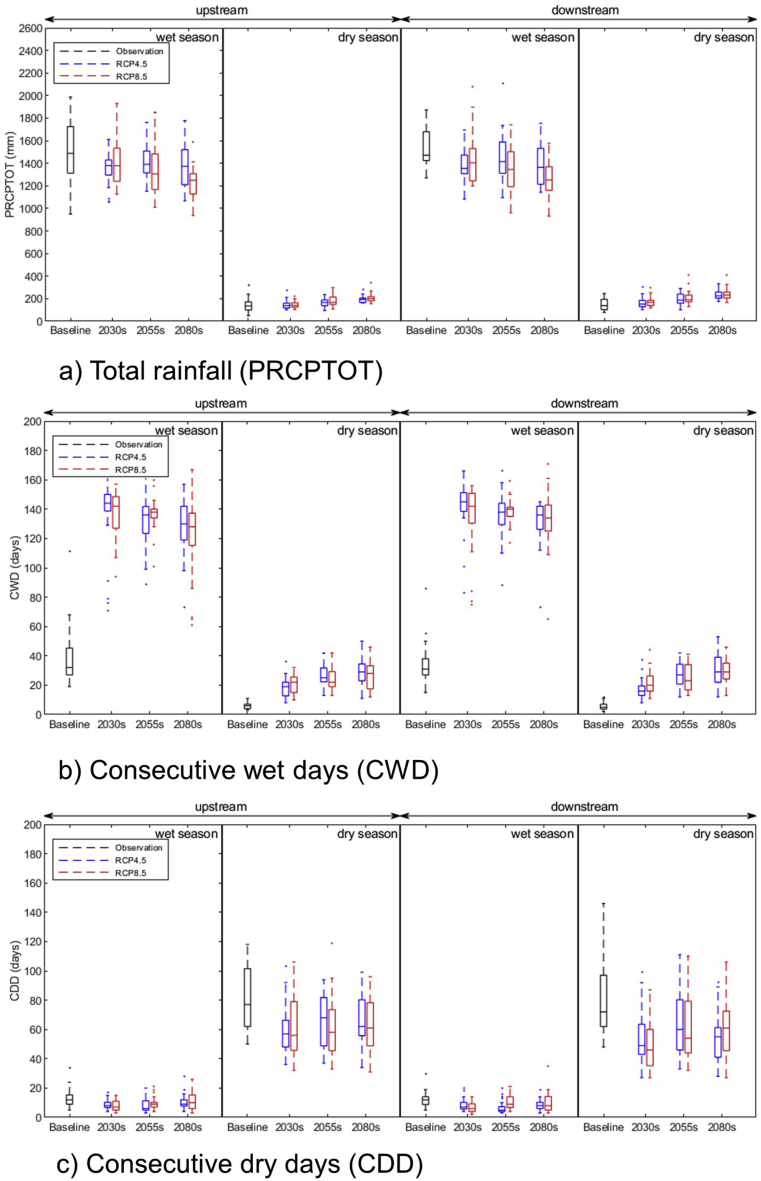
(a) projected total rainfall, (b) consecutive wet days, and (c) consecutive dry days upstream and downstream of the LSRB during wet and dry seasons under RCP4.5 and RCP8.5 scenarios.

In both upstream and downstream areas of the basin, consecutive dry days (CDD) are expected to decrease and consecutive wet days (CWD) increase in the future (Figure 8b and 8c). The seasonal total rainfall (PRCPTOT) is observed to increase in the dry season and decrease in the wet in both areas (Figure 8a). During the dry season, there are approximately 80 consecutive dry days and 12 days in the wet season, and these figures are expected to reduce by at least 22% in the far future, with a higher percentage reduction in the near future and a lower percentage reduction in the far future (Table 10). The baseline average precipitation in the wet season of approximately 1500 mm is expected to decrease to 1220–1380 mm in the far future. On the other hand, total precipitation in the dry season of approximately 145 mm is expected to increase to more than 200 mm, with a higher increase in downstream areas (Table 10).

**Table 10:** Comparison of future extreme rainfall indices with the baseline period during wet and dry seasons upstream and downstream of the LSRB. The value represents an ensemble of three RCMs.

Indices	Period	Upstream				Downstream			
		Wet season		Dry season		Wet season		Dry season	
		RCP4.5	RCP8.5	RCP4.5	RCP8.5	RCP4.5	RCP8.5	RCP4.5	RCP8.5
CDD (days)	Baseline	13		81		12		80	
	2030s	9	8	59	62	9	7	55	50
	change	-5	-6	-22	-20	-3	-5	-25	-30
	2055s	8	10	67	62	7	10	64	62
	change	-5	-4	-14	-19	-5	-1	-16	-18
	2080s	10	11	67	62	8	10	54	62
	change	-3	-2	-14	-19	-3	-2	-26	-18
CWD (days)	Baseline	39		6		34		6	
	2030s	136	136	18	21	141	135	17	22
	change	97	97	12	15	107	101	12	16
	2055s	133	137	27	25	135	139	27	25
	change	94	97	21	19	101	105	22	19
	2080s	127	124	28	26	131	132	30	29
	change	88	85	22	20	97	98	24	23
PRCPTOT (mm)	Baseline	1498		145		1531		150	
	2030s	1353	1410	144	148	1377	1449	162	172
	change	-145	-88	-1	3	-154	-82	12	22
	2055s	1407	1346	167	182	1433	1362	200	210
	change	-91	-152	22	37	-98	-169	50	60
	2080s	1380	1223	192	207	1389	1248	229	242
	change	-118	-275	47	62	-142	-283	80	92
R95p (mm)	Baseline	306		4		319		2	
	2030s	340	408	11	11	318	397	14	16
	change	35	102	7	7	-1	78	12	15
	2055s	388	373	8	18	385	340	11	22
	change	82	67	4	14	66	20	9	20
	2080s	425	264	8	13	399	264	14	23
	change	119	-42	4	9	80	-55	12	21
RX1day (mm)	Baseline	48		20		51		21	
	2030s	74	84	17	16	60	71	17	19
	change	26	36	-3	-4	9	20	-4	-2
	2055s	69	70	14	20	74	65	18	20
	change	21	22	-6	0	23	13	-3	-1
	2080s	83	67	16	18	68	60	18	23
	change	35	19	-4	-2	16	9	-3	2
RX5day (mm)	Baseline	142		44		142		42	
	2030s	153	173	31	32	149	160	33	38
	change	12	32	-13	-13	7	18	-9	-5
	2055s	159	144	30	40	173	139	40	42
	change	18	3	-14	-4	31	-3	-2	0
	2080s	193	141	32	38	172	130	40	46
	change	51	-1	-12	-6	30	-12	-2	4
R20 (days)	Baseline	22		1		23		1	
	2030s	9	10	0	0	9	10	0	0
	change	-13	-12	-1	-1	-15	-13	-1	-1
	2055s	10	9	0	0	10	9	0	1
	change	-11	-13	-1	0	-13	-15	-1	0
	2080s	11	6	0	0	10	7	1	1
	change	-11	-15	-1	-1	-13	-17	0	0

## 4 Conclusions

This study presents the results of projected future changes in extreme temperature and rainfall events over the Lower Songkhram River Basin in Thailand, prepared using an ensemble of three RCMs under two RCP scenarios. The results suggest that the average annual and monthly maximum and minimum temperatures in the basin are projected to increase in future, with a lesser increase in the near and a greater increase in the far future. Similarly, the basin is expected to be warmer with increasing heat events and decreasing cold events during wet and dry seasons at both upstream and downstream locations. The future average annual rainfall in the basin is projected to decrease. However, variability in average monthly rainfall is expected to increase in the dry season (Jan–May) and decrease in the wet (Aug–Dec). The most intense rainfall in one day and five consecutive days in the wet season is observed to increase in future, with a higher increase in the near future and a lower increase in the far future. Very heavy rainfall days (the number of days receiving more than 20 mm/day of rainfall in the basin) are observed to be decreasing in both wet and dry seasons at the two locations. The projections show the expected range of changes in rainfall and temperature from the outputs of three RCMs. However, it is important to note that these projections may contain uncertainties and are further limited by the use of outputs from a smaller number of RCMs and only two scenarios as well as the small number of meteorological stations in the study area. Therefore, the values should be carefully interpreted for further use.

## Declarations

### Author contribution statement

S. Shrestha: Performed the experiments; Contributed reagents, materials, analysis tools or data; Wrote the paper.

R. Roachanakanan: Conceived and designed the experiments; Analyzed and interpreted the data; Wrote the paper.

### Funding statement

This research did not receive any specific grant from funding agencies in the public, commercial, or not-for-profit sectors.

### Declaration of interests statement

The authors declare no conflict of interest.

### Additional information

No additional information is available for this paper.

## Appendix A Supplementary data

Supplementary_Information_CC-LSRB_Revised_V2 Supplementary_Information_CC-LSRB_Revised_V2
