# Supplementary material for: Extreme climate projections under representative concentration pathways in the Lower Songkhram River Basin, Thailand
Source: Heliyon. 2021 Feb 16;7(2):e06146. doi: 10.1016/j.heliyon.2021.e06146 (PMC7900689; doi:10.1016/j.heliyon.2021.e06146)
Supplement: Supplementary_Information_CC-LSRB_Revised_V2 [file mmc1.docx]

Supplementary Information for

**Extreme climate projections under representative concentration pathways in the Lower Songkhram River Basin, Thailand**

Sumana Shrestha*, Raywadee Roachanakanan

Faculty of Environment and Resource Studies, Mahidol University,

Salaya Campus, Nakhon Pathom 73170, Thailand

**Study Area**


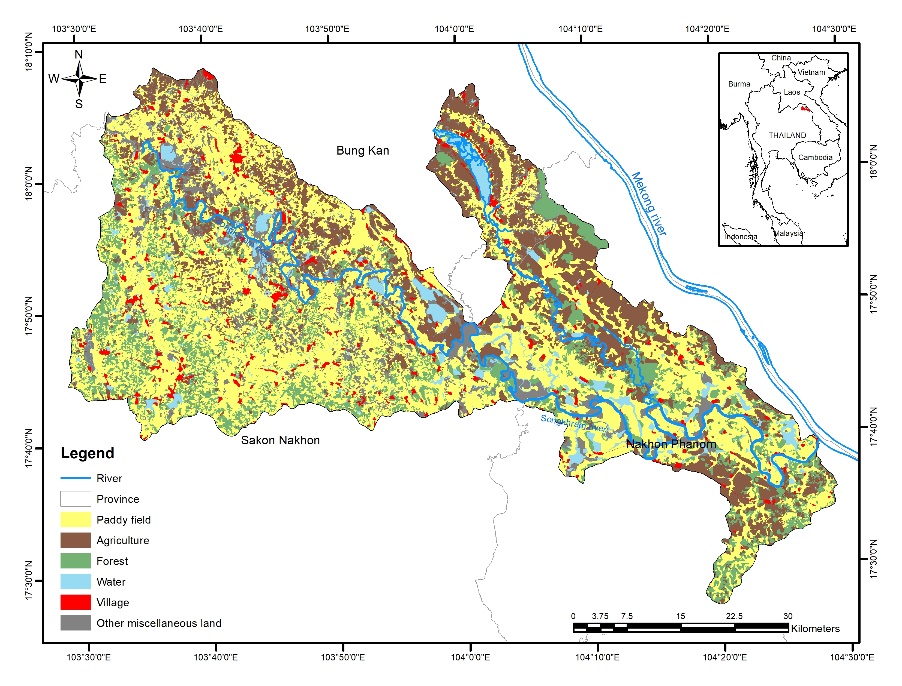


Figure S1. Land use and land cover of the Lower Songkhram River Basin, Thailand (LDD, 2014)

**Performance evaluation of bias correction**

Table S1. Comparison of mean and SD of maximum temperature of RCM and observed data

| **Station** |  | **ACCESS1-CSIRO-CCAM** | | **CNRM-CM5-CSIRO-CCAM** | | **MPI-ESM-LR-CSIRO-CCAM** | |
| --- | --- | --- | --- | --- | --- | --- | --- |
|  |  | **Mean** | **SD (°C)** | **Mean** | **SD (°C)** | **Mean** | **SD (°C)** |
| Nakhon Phanom | Obs | 31.7 | 3.1 | 31.7 | 3.1 | 31.7 | 3.1 |
|  | RCM His | 29.1 | 5.5 | 29.0 | 5.6 | 29.4 | 5.4 |
|  | RCM Corr His | 31.7 | 4.6 | 31.7 | 4.5 | 31.7 | 4.6 |
| Nakhon Phanom Agromet | Obs | 30.8 | 3.2 | 30.8 | 3.2 | 30.8 | 3.2 |
|  | RCM His | 29.1 | 5.5 | 29.0 | 5.6 | 29.4 | 5.4 |
|  | RCM Corr His | 30.8 | 4.6 | 30.8 | 4.5 | 30.8 | 4.6 |
| Nong Khai | Obs | 32.2 | 3.3 | 32.2 | 3.3 | 32.2 | 3.3 |
|  | RCM His | 31.3 | 4.0 | 31.1 | 4.1 | 31.3 | 4.0 |
|  | RCM Corr His | 32.2 | 3.8 | 32.2 | 3.8 | 32.2 | 3.8 |
| Sakon Nakhon | Obs | 31.6 | 3.2 | 31.6 | 3.2 | 31.6 | 3.2 |
|  | RCM His | 31.0 | 4.3 | 30.8 | 4.4 | 31.1 | 4.3 |
|  | RCM Corr His | 31.6 | 4.0 | 31.6 | 3.9 | 31.6 | 4.0 |
| Sakon Nakhon Agromet | Obs | 31.7 | 3.1 | 31.7 | 3.1 | 31.7 | 3.1 |
|  | RCM His | 31.0 | 4.3 | 30.8 | 4.3 | 31.1 | 4.2 |
|  | RCM Corr His | 31.7 | 3.9 | 31.7 | 3.8 | 31.7 | 3.8 |
| Udon Thani | Obs | 32.4 | 3.2 | 32.4 | 3.2 | 32.4 | 3.2 |
|  | RCM His | 31.3 | 4.0 | 31.1 | 4.1 | 31.3 | 4.0 |
|  | RCM Corr His | 32.4 | 3.8 | 32.4 | 3.9 | 32.4 | 3.8 |

Table S2. Comparison of mean and SD of minimum temperature of RCM and observed data

| **Station** |  | **ACCESS1-CSIRO-CCAM** | | **CNRM-CM5-CSIRO-CCAM** | | **MPI-ESM-LR-CSIRO-CCAM** | |
| --- | --- | --- | --- | --- | --- | --- | --- |
|  |  | **Mean** | **SD (°C)** | **Mean** | **SD (°C)** | **Mean** | **SD (°C)** |
| Nakhon Phanom | Obs | 21.6 | 3.8 | 21.6 | 3.8 | 21.6 | 3.8 |
|  | RCM His | 20.4 | 4.1 | 20.3 | 4.3 | 20.4 | 4.1 |
|  | RCM Corr His | 21.6 | 4.0 | 21.6 | 4.0 | 21.6 | 4.1 |
| Nakhon Phanom Agromet | Obs | 20.6 | 4.2 | 20.6 | 4.2 | 20.6 | 4.2 |
|  | RCM His | 20.4 | 4.1 | 20.3 | 4.3 | 20.4 | 4.1 |
|  | RCM Corr His | 20.6 | 4.4 | 20.6 | 4.4 | 20.6 | 4.4 |
| Nong Khai | Obs | 21.9 | 3.7 | 21.9 | 3.7 | 21.9 | 3.7 |
|  | RCM His | 21.9 | 4.4 | 21.7 | 4.6 | 21.9 | 4.5 |
|  | RCM Corr His | 21.9 | 4.1 | 21.9 | 4.1 | 21.9 | 4.1 |
| Sakon Nakhon | Obs | 21.9 | 4.0 | 21.9 | 4.0 | 21.9 | 4.0 |
|  | RCM His | 21.7 | 4.5 | 21.5 | 4.7 | 21.7 | 4.5 |
|  | RCM Corr His | 21.9 | 4.3 | 21.9 | 4.3 | 21.9 | 4.3 |
| Sakon Nakhon Agromet | Obs | 20.9 | 4.3 | 20.9 | 4.3 | 20.9 | 4.3 |
|  | RCM His | 21.9 | 4.2 | 21.7 | 4.4 | 21.8 | 4.2 |
|  | RCM Corr His | 20.9 | 4.5 | 20.9 | 4.5 | 20.9 | 4.5 |
| Udon Thani | Obs | 22.0 | 3.8 | 22.0 | 3.8 | 22.0 | 3.8 |
|  | RCM His | 21.9 | 4.4 | 21.7 | 4.6 | 21.9 | 4.5 |
|  | RCM Corr His | 22.0 | 4.1 | 22.0 | 4.2 | 22.0 | 4.2 |

Table S3. Comparison of mean and SD rainfall of RCM and observed data

| **Station** |  | **ACCESS1-CSIRO-CCAM** |  | **CNRM-CM5-CSIRO-CCAM** |  | **MPI-ESM-LR-CSIRO-CCAM** |  |
| --- | --- | --- | --- | --- | --- | --- | --- |
|  |  | **Mean** | **SD (mm)** | **Mean** | **SD (mm)** | **Mean** | **SD (mm)** |
| A. Phon Charoen | Obs | 1810 | 171 | 1810 | 171 | 1810 | 171 |
|  | RCM His | 620 | 51 | 656 | 65 | 639 | 56 |
|  | RCM Corr His | 1810 | 185 | 1810 | 196 | 1810 | 185 |
| A. Seka | Obs | 1901 | 159 | 1901 | 159 | 1901 | 159 |
|  | RCM His | 620 | 51 | 656 | 65 | 639 | 56 |
|  | RCM Corr His | 1901 | 187 | 1901 | 199 | 1901 | 189 |
| A. So Pisai | Obs | 1665 | 137 | 1665 | 137 | 1665 | 137 |
|  | RCM His | 706 | 63 | 761 | 77 | 724 | 67 |
|  | RCM Corr His | 1665 | 165 | 1665 | 184 | 1665 | 168 |
| A. Ban Muang | Obs | 1851 | 161 | 1851 | 161 | 1851 | 161 |
|  | RCM His | 615 | 46 | 650 | 60 | 621 | 47 |
|  | RCM Corr His | 1851 | 171 | 1851 | 184 | 1851 | 170 |
| A. Wanon Niwat | Obs | 1450 | 119 | 1450 | 119 | 1450 | 119 |
|  | RCM His | 620 | 51 | 656 | 65 | 639 | 56 |
|  | RCM Corr His | 1450 | 140 | 1450 | 148 | 1450 | 140 |
| A. Na Thom | Obs | 1452 | 116 | 1452 | 116 | 1452 | 116 |
|  | RCM His | 620 | 51 | 656 | 65 | 639 | 56 |
|  | RCM Corr His | 1452 | 138 | 1452 | 144 | 1452 | 137 |
| A. Si Songkhram | Obs | 1578 | 131 | 1578 | 131 | 1578 | 131 |
|  | RCM His | 604 | 48 | 639 | 60 | 610 | 54 |
|  | RCM Corr His | 1578 | 148 | 1578 | 162 | 1578 | 161 |
| A. Tha Uthen | Obs | 2295 | 203 | 2295 | 203 | 2295 | 203 |
|  | RCM His | 585 | 56 | 600 | 61 | 551 | 49 |
|  | RCM Corr His | 2295 | 244 | 2295 | 249 | 2295 | 238 |
